# Single-nucleus transcriptome analysis reveals dysregulation of angiogenic endothelial cells and neuroprotective glia in Alzheimer’s disease

**DOI:** 10.1073/pnas.2008762117

**Published:** 2020-09-28

**Authors:** Shun-Fat Lau, Han Cao, Amy K. Y. Fu, Nancy Y. Ip

**Affiliations:** ^a^Division of Life Science, State Key Laboratory of Molecular Neuroscience, Molecular Neuroscience Center, The Hong Kong University of Science and Technology, Hong Kong, China;; ^b^Hong Kong Center for Neurodegenerative Diseases, Hong Kong, China;; ^c^Guangdong Provincial Key Laboratory of Brain Science, Disease and Drug Development, Hong Kong University of Science and Technology Shenzhen Research Institute, Shenzhen–Hong Kong Institute of Brain Science, 518057 Shenzhen, Guangdong, China

**Keywords:** synaptic signaling, synapse, neurodegenerative diseases, myelination, angiogenesis

## Abstract

Alzheimer’s disease (AD) is the most common form of dementia but has no effective treatment. A comprehensive investigation of cell type-specific responses and cellular heterogeneity in AD is required to provide precise molecular and cellular targets for therapeutic development. Accordingly, we perform single-nucleus transcriptome analysis of 169,496 nuclei from the prefrontal cortical samples of AD patients and normal control (NC) subjects. Differential analysis shows that the cell type-specific transcriptomic changes in AD are associated with the disruption of biological processes including angiogenesis, immune activation, synaptic signaling, and myelination. Subcluster analysis reveals that compared to NC brains, AD brains contain fewer neuroprotective astrocytes and oligodendrocytes. Importantly, our findings show that a subpopulation of angiogenic endothelial cells is induced in the brain in patients with AD. These angiogenic endothelial cells exhibit increased expression of angiogenic growth factors and their receptors (i.e., *EGFL7*, *FLT1*, and *VWF*) and antigen-presentation machinery (i.e., *B2M* and *HLA-E*). This suggests that these endothelial cells contribute to angiogenesis and immune response in AD pathogenesis. Thus, our comprehensive molecular profiling of brain samples from patients with AD reveals previously unknown molecular changes as well as cellular targets that potentially underlie the functional dysregulation of endothelial cells, astrocytes, and oligodendrocytes in AD, providing important insights for therapeutic development.

## Significance

Alzheimer’s disease (AD) has complex pathological mechanisms. Our limited understanding of the molecular and cellular changes in AD hindered the identification of therapeutic targets. In this study, we profiled 169,496 nuclei from the prefrontal cortical samples of AD patients and healthy controls by single-nucleus RNA sequencing. The results indicated that there is a loss of neuroprotective glial cells in AD, which may contribute to impaired neurotransmitter recycling in astrocytes, demyelination in oligodendrocytes, and perturbed synaptic pruning in microglia. Moreover, the analysis revealed a role of antigen presentation by angiogenic endothelial cells in AD. Together, our findings offer important insights into the therapeutic potential of targeting glial- and endothelial-specific pathways to restore brain homeostasis in AD.

The global prevalence of Alzheimer’s disease (AD), which is the most common form of dementia, is currently 24 million and is expected to double every 20 years (1). Its pathological hallmarks include the deposition of amyloid-beta peptides and neurofibrillary tangles as well as neuroinflammation (2). However, there is still no effective treatment for AD, which is in part because of the incomplete understanding of the molecular basis of cell type-specific responses during AD pathogenesis, including impaired synaptic functions (3–6), the loss of blood–brain barrier protection, and the loss of neurotrophic support (7–11), respectively.

Along AD progression, the disruption of the molecular pathways of specific cell types can contribute to their observed dysfunctions. Recent studies utilized single-nucleus transcriptome analysis to investigate the transcriptomic changes in AD brains (12–14) and have revealed molecular alterations at the single-cell level using readily available frozen brain tissues. In particular, such studies identified the dysregulated pathways in the most predominant neural cell types, such as neurons and oligodendrocytes, in AD. Although the data from these studies are valuable resources for researchers, some issues remain unresolved. First, the dysregulated pathways in other cell types, particularly endothelial cells, remain unknown. Second, because of cellular heterogeneity, it is unclear whether and how changes in cell subpopulations in AD contribute to the observed dysregulations. Therefore, it is essential to investigate the changes in cellular heterogeneity in AD in order to identify precise cellular targets for AD therapeutic development.

Accordingly, in order to comprehensively investigate the dysregulated molecular pathways in different cell types in AD and determine the cellular targets that contribute to the observed changes, we performed single-nucleus transcriptome analysis of the AD brain by profiling 169,496 nuclei from AD patients and healthy normal control (NC) subjects. Our unbiased transcriptome analysis showed that the AD-related, cell type-specific transcriptomic changes in endothelial cells, astrocytes, and oligodendrocytes are associated with the dysregulation of their respective functions, namely angiogenesis, synaptic signaling, and myelination. Subcluster analysis further identified that the induction of angiogenic endothelial cells and reduction of neuroprotective astrocytes and oligodendrocytes contribute to the transcriptomic changes in AD. In particular, the transcriptomic signature of angiogenic endothelial cells revealed their association with antigen presentation, suggesting that the dysregulated angiogenesis and antigen presentation in endothelial cells potentially contribute to AD pathogenesis. Hence, our comprehensive transcriptome profiling of brain tissues from AD patients provides important insights that could aid both prognostic evaluation and therapeutic targeting of endothelial- or glial-specific pathways in AD.

## Results

### Single-Nucleus Transcriptome Profiling of the Prefrontal Cortex in AD.

**Fig. 1.**
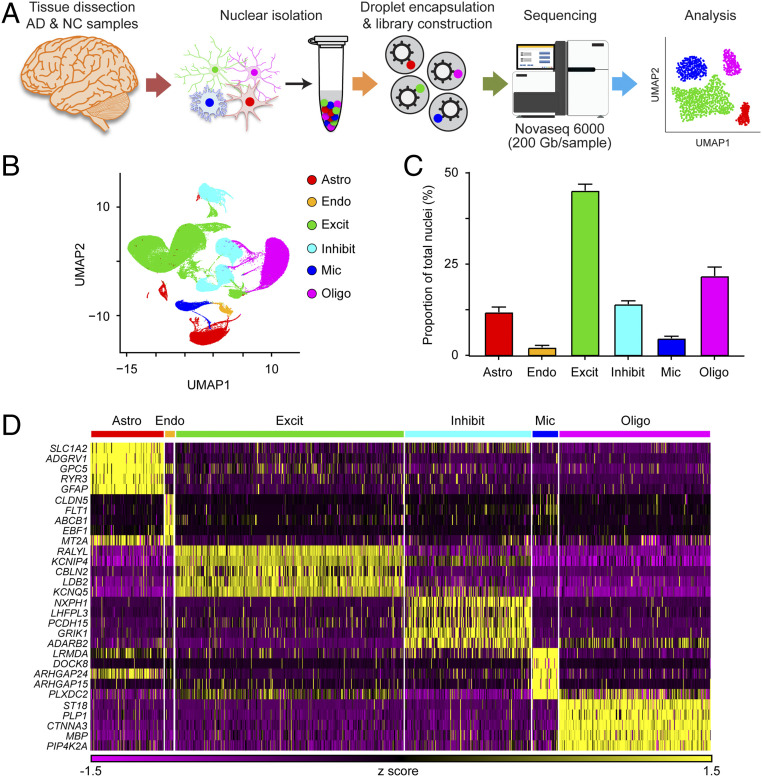
Single-nucleus transcriptome analysis of the prefrontal cortex in AD. (*A*) Single-nucleus transcriptome profiling workflow. (*B*–*D*) Unbiased identification of cell-type heterogeneity in the human prefrontal cortex. (*B*) UMAP plot showing the six major cell types isolated from prefrontal cortex. (*C*) Proportions of cell types among the 169,496 sampled nuclei. (*D*) Heatmap showing the top five most enriched genes for each cell type. All data are mean ± SEM. See also *SI Appendix*, Fig. S1 and Tables S1 and S2.

To investigate how the molecular and cellular profiles of brain tissues are altered in patients with AD compared to those in healthy NC subjects, we performed transcriptome analysis of 21 prefrontal cortex tissue samples from patients with AD (*n* = 12) and NC subjects (*n* = 9) at the single-cell level by single-nucleus RNA sequencing (snRNA-seq). A diagram of the experimental methodology is shown in Fig. 1*A*, and the sequencing characteristics of the samples are shown in *SI Appendix*, Table S1. We sampled 169,496 nuclei: 90,713 and 78,783 nuclei from AD and NC brain samples, respectively. Despite the variations in postmortem delay among samples, we showed that the sample quality—indicated by the mean numbers of transcripts and genes detected per nucleus—did not seem to be affected by the postmortem delay (*SI Appendix*, Fig. S1*A*). In addition, these parameters were comparable to those reported in a previous snRNA-seq study (15), confirming the quality of our samples prior to downstream analysis.

To establish a baseline profile of cell populations, we performed initial unbiased uniform manifold approximation and projection (UMAP) clustering on all 21 samples from both AD and NC subjects. This analysis yielded 43 unique cell clusters (*SI Appendix*, Fig. S1*B*), which we subsequently categorized into the following six major cell types according to their individual transcriptome profiles and previously reported cell-type markers: astrocytes (*AQP4*^+^, 11.9 ± 1.4% of total nuclei), endothelial cells (*CLDN5*^+^, 2.3 ± 0.5%), excitatory neurons (*CAMK2A*^+^, 45.2 ± 1.7%), inhibitory neurons (*GAD1*^+^, 14.1 ± 0.9%), microglia (*C3*^+^, 4.7 ± 0.6%), and oligodendrocytes (*MBP*^+^, 21.8 ± 2.5%) (Fig. 1 *B* and *C* and *SI Appendix*, Fig. S1 *C* and *D*). In addition to the previously reported cell-type markers, these six major cell types expressed the following unique signature genes, which can serve as novel cell-type markers: *ADGRV1*, *GPC5*, and *RYR3* were expressed by astrocytes; *ABCB1* and *EBF1* by endothelial cells; *CBLN2* and *LDB2* by excitatory neurons; *LHFPL3* and *PCDH15* by inhibitory neurons; *LRMDA* and *DOCK8* by microglia; and *PLP1* and *ST18* by oligodendrocytes (Fig. 1*D* and *SI Appendix*, Table S2). These results collectively and comprehensively reveal the cell-type heterogeneity in diseased and healthy brain tissues.

### Cell Type-Specific Transcriptomic Changes Reveal Dysregulated Molecular Pathways in AD Brains.

**Fig. 2.**
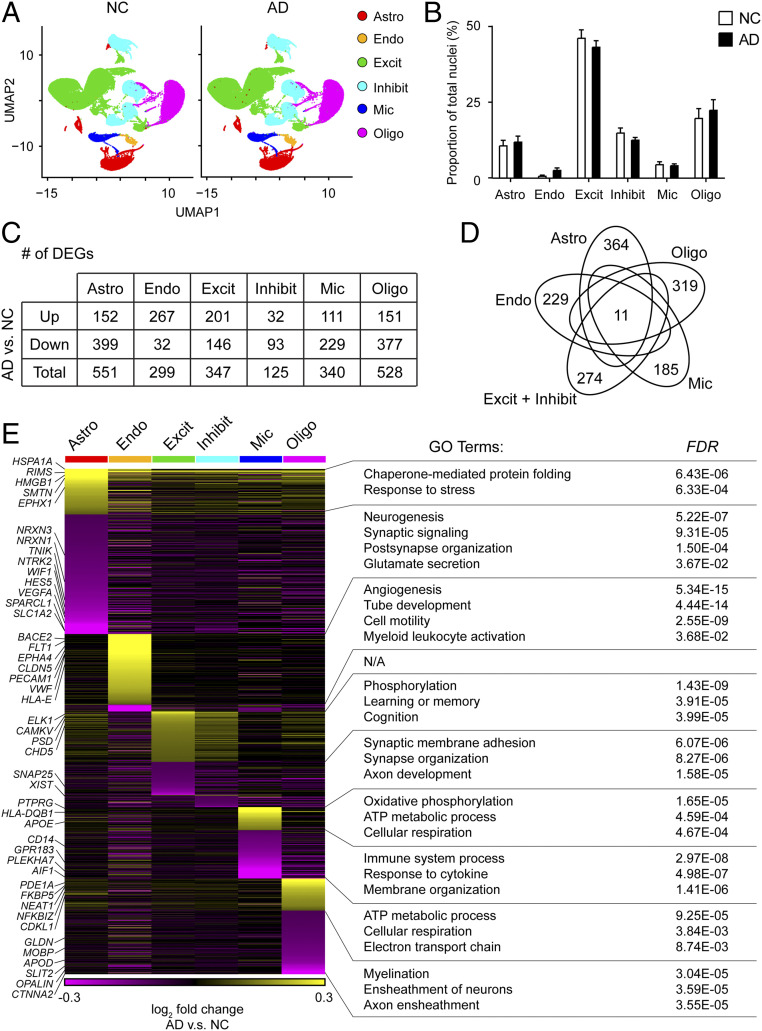
Dysregulated molecular pathways in AD according to cell type-specific transcriptomic changes. UMAP plots (*A*) and bar plot (*B*) showing the proportions of the six major cell types found in the AD and NC prefrontal cortical samples. (*C* and *D*) AD-associated transcriptomic changes were highly cell type-specific. (*C*) Numbers of DEGs between AD and NC samples within each cell type (adjusted *P* < 0.1, log_2_ fold change ≥ 0.1 or ≤ −0.1). Down: down-regulated; Up: up-regulated. (*D*) Venn diagram showing the 11 coregulated DEGs among all six cell types. Also shown are the numbers of DEGs specific to each cell type. (*E*) The cell type-specific transcriptomic changes in AD were associated with distinct molecular pathways. Heatmap showing the expression changes of DEGs in all six cell types in AD samples. Specific GO terms are listed at *Right*. Astro: astrocytes, Endo: endothelial cells, Excit: excitatory neurons, Inhibit: inhibitory neurons, Mic: microglia, and Oligo: oligodendrocytes. All data are mean ± SEM. See also *SI Appendix*, Figs. S2 and S4 and Table S3.

Following our initial cell-type characterization, we compared the proportions of different cell types between AD and NC brain samples. UMAP cluster analysis revealed that the proportions of astrocytes, excitatory neurons, inhibitory neurons, microglia, and oligodendrocytes were similar between the AD and NC brain samples. However, the proportion of endothelial cells was higher in the AD samples than the NC samples (3.0 ± 0.9% vs. 1.2 ± 0.3%, respectively; Fig. 2 *A* and *B*).

To examine the global transcriptomic changes in individual brain cell types in AD, we compared the individual cell-type transcriptome profiles between AD and NC samples. We identified the following 2,190 differentially expressed genes (DEGs) between the AD and NC brain samples: 551 in astrocytes, 299 in endothelial cells, 347 in excitatory neurons, 125 in inhibitory neurons, 340 in microglia, and 528 in oligodendrocytes (Fig. 2*C* and *SI Appendix*, Table S3). Among the 2,190 DEGs, only 11 were differentially expressed in all cell types (Fig. 2*D*), suggesting that most observed AD-associated transcriptomic changes are cell type-specific.

As changes in molecular phenotypes provide insights into the functional changes in individual cell types, we functionally annotated the DEGs associated with AD in individual cell types by performing pathway analysis (Fig. 2*E* and *SI Appendix*, Fig. S2). Our findings demonstrated that the DEGs in both excitatory and inhibitory neurons (e.g., *SNAP25*) were associated with synaptic organization and adhesion. Similarly, the down-regulated genes in astrocytes (e.g., *HES5*, *NTRK2*, *SLC1A2*, *SPARCL1*, and *WIF1*) were associated with synaptic signaling and glutamate secretion. Specifically, the expression levels of astrocytic neurexin genes (including *NRXN1* and *NRXN3*), which regulate excitatory synaptogenesis (16, 17), were down-regulated in the AD samples.

Besides the transcriptomic changes in neurons and astrocytes, our pathway analysis also revealed that the DEGs in microglia (e.g., *AIF1*, *CD14*, *GPR183*, and *PLEKHA7*), endothelial cells (e.g., *CLDN5*, *EPHA4*, *FLT1*, *PECAM1*, and *VWF*), and oligodendrocytes (e.g., *CTNNA2* and *OPALIN*) were associated with immune response, angiogenesis, and myelination, respectively (Fig. 2*E*). Of note, endothelial cells also exhibited dysregulation of immune response-related genes (e.g., *HLA-E*) in AD (Fig. 2*E*).

Together, our results reveal that the cell type-specific transcriptomic changes in AD are associated with four molecular pathways: angiogenesis in endothelial cells, immune response in endothelial cells and microglia, myelination in oligodendrocytes, and synaptic signaling in astrocytes and neurons (Fig. 2*E*).

### Cross-Study Validation of the Transcriptomic Changes in AD Brains.

**Fig. 3.**
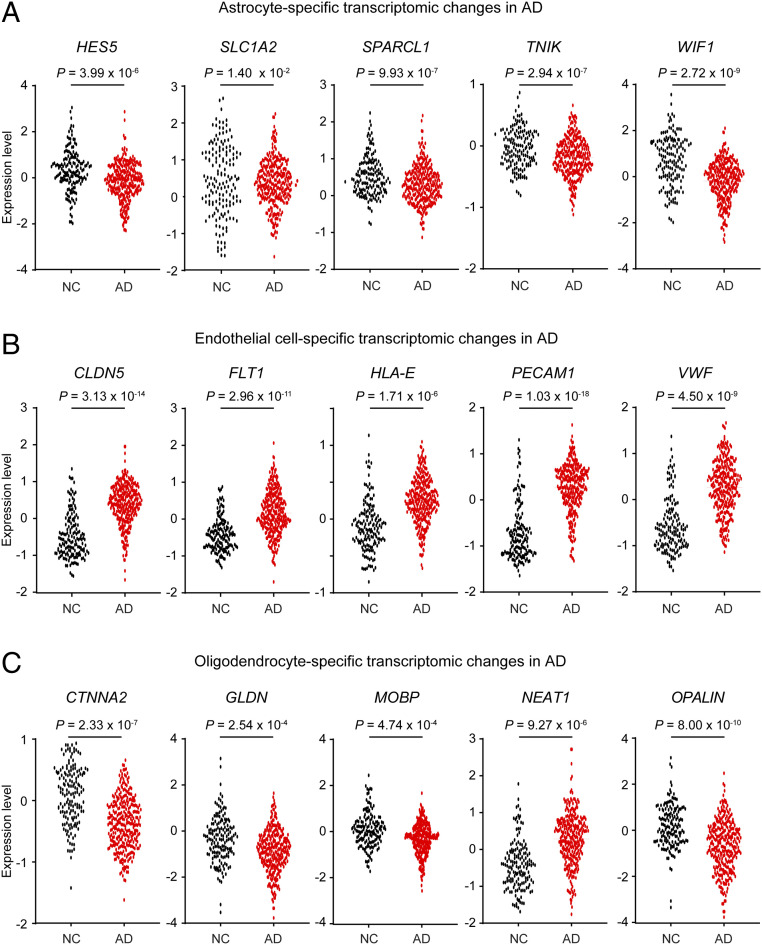
Validation of the cell type-specific transcriptomic changes in AD based on data from a large cohort microarray study. Dot plots showing the expression levels of DEGs specific to astrocytes (*A*), endothelial cells (*B*), and oligodendrocytes (*C*) in the prefrontal cortex from patients with AD (*n* = 310) and NC (*n* = 157) samples. Each dot represents the expression level from an individual subject. Source: Narayanan et al. (18). See also *SI Appendix*, Fig. S3 and Table S4.

To validate the AD-associated transcriptomic changes detailed above, we compared our results with microarray data from large cohort studies that examined samples from the prefrontal cortex (AD: *n* = 310; NC: *n* = 157) or temporal cortex (AD: *n* = 106; NC: *n* = 135) (*SI Appendix*, Table S4) (18, 19). Among the DEGs identified in our snRNA-seq analysis, 1,113 and 764 genes were significantly differentially expressed in the microarray data from the prefrontal cortex and temporal cortex, respectively (adjusted *P* < 0.05) (*SI Appendix*, Table S4). In addition, 86.7% of the replicable DEGs in the prefrontal cortex microarray exhibited concordant changes in expression levels; these included changes specific to astrocytes (e.g., *HES5*, *SLC1A2*, *SPARCL1*, *TNIK*, and *WIF1*), endothelial cells (e.g., *CLDN5*, *FLT1*, *HLA-E*, *PECAM1*, and *VWF*), and oligodendrocytes (e.g., *CTNNA2*, *GLDN*, *MOBP*, *NEAT1*, and *OPALIN*) (Fig. 3 *A*–*C*). Stratification of the expression levels of these DEGs by sex showed that their expression changes were concordant in both sexes but to different degrees (*SI Appendix*, Fig. S3). These results validate the AD-associated transcriptomic changes identified in our snRNA-seq analysis.

While comparison with bulk microarray data validated the transcriptomic changes observed in AD brains at the global transcriptome level, the bulk data were unable to distinguish cell type-specific changes. Therefore, we compared our findings with the snRNA-seq data reported by Mathys et al., who analyzed gene expression in the prefrontal cortex of patients with various degrees of AD pathology (*SI Appendix*, Fig. S4) (12). We found 273 cell type-specific DEGs in common between the two datasets, including 53 in astrocytes, 97 in excitatory neurons, 24 in inhibitory neurons, 13 in microglia, and 86 in oligodendrocytes (*SI Appendix*, Fig. S4 *A*, *B*, *D*, *E*, *G*, *H*, *J*, *K*, *M*, and *N*). More than 90% of the overlapping genes, especially the astrocyte-specific and neuron-specific DEGs, exhibited concordant changes in AD (*SI Appendix*, Fig. S4 *B*, *E*, and *H*). Moreover, pathway analysis showed that these overlapping DEGs were associated with transsynaptic signaling in astrocytes (*SI Appendix*, Fig. S4*C*), synaptic signaling in excitatory neurons (*SI Appendix*, Fig. S4*F*), mitochondrial functions in inhibitory neurons (*SI Appendix*, Fig. S4*I*), secretion in microglia (*SI Appendix*, Fig. S4*L*), and axonogenesis in oligodendrocytes (*SI Appendix*, Fig. S4*O*). Thus, these findings validate the cell type-specific dysregulated pathways identified in our study.

### The Disrupted Subpopulation Heterogeneity of Astrocytes and Oligodendrocytes Contributes to Neuronal Dysfunction in AD.

**Fig. 4.**
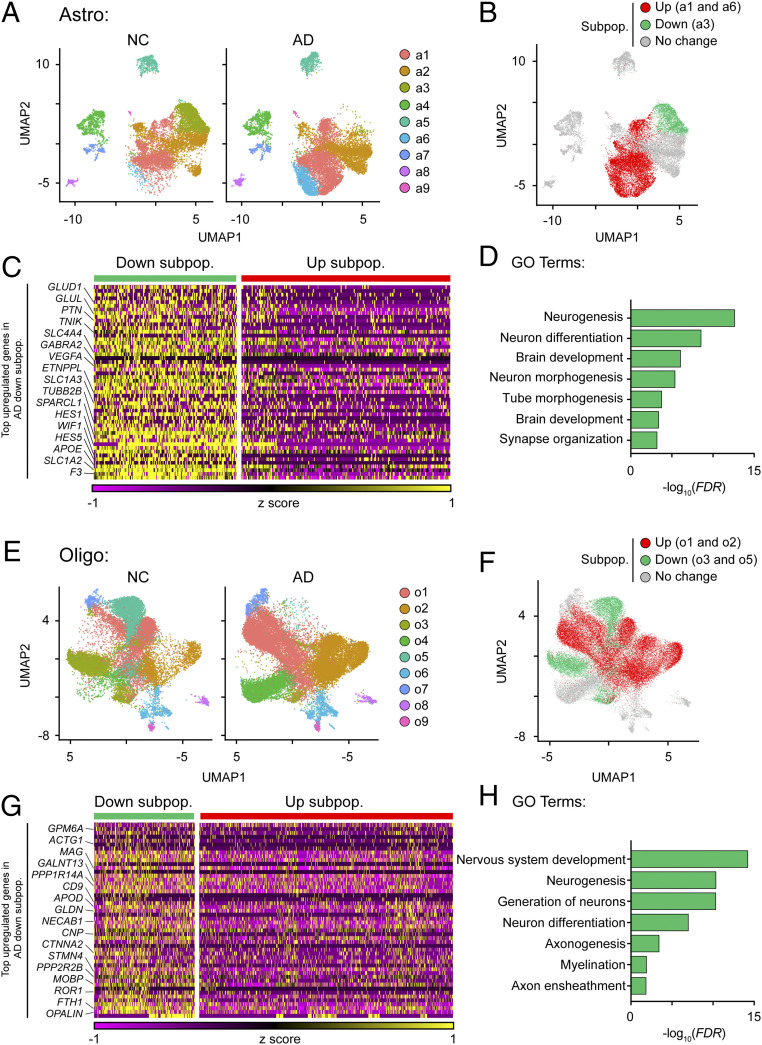
The proportions of astrocytes and oligodendrocytes that regulate neuronal homeostasis are reduced in AD. (*A*) UMAP plots showing the distributions of astrocyte subpopulations (a1–a9) in AD and NC prefrontal cortical samples. (*B*–*D*) The astrocyte subpopulation associated with the maintenance of synaptic functions was reduced in AD. (*B*) UMAP plot showing the distribution of the AD-associated astrocyte subpopulations. (*C*) Heatmap showing the expression levels of the top enriched genes in the AD-down-regulated astrocyte subpopulation (adjusted *P* < 0.1, log_2_ fold change ≥ 0.1). Down subpop.: down-regulated subpopulation; Up subpop.: up-regulated subpopulation. (*D*) GO pathway analysis of the transcriptomic signature of the AD-down-regulated astrocyte subpopulation. (*E*) UMAP plots showing the distributions of oligodendrocyte subpopulations (o1–o9) in AD and NC prefrontal cortical samples. (*F*–*H*) Oligodendrocyte subpopulations associated with myelination were reduced in AD. (*F*) UMAP plot showing the distribution of the AD-associated oligodendrocyte subpopulations. (*G*) Top enriched genes in the AD-down-regulated oligodendrocyte subpopulations (adjusted *P* < 0.1, log_2_ fold change ≥ 0.1). Down subpop.: down-regulated subpopulation; Up subpop.: up-regulated subpopulation. (*H*) Pathway analysis of the transcriptomic signature of the AD-down-regulated oligodendrocyte subpopulations. See also *SI Appendix*, Figs. S5 and S6 and Table S5.

Next, we investigated the subpopulation heterogeneity within individual cell types in AD and NC samples. To this end, we performed subcluster analysis of individual cell types (except excitatory and inhibitory neurons, which exhibited only subtle changes in DEG expression levels). Subcluster analysis of astrocytes identified nine transcriptomically unique subpopulations (Fig. 4*A*). The relative proportions of subpopulations a2, a4, a5, a7, a8, and a9 were similar between the AD and NC samples (Fig. 4*A* and *SI Appendix*, Fig. S5*A*). However, compared to the NC samples, the relative proportions of a1 and a6 were 9.9% and 10.2% larger in the AD samples, respectively, while that of a3 was 23.5% smaller (Fig. 4*A* and *SI Appendix*, Fig. S5*A*). Only subpopulations a1, a3, and a6 expressed DEGs; specifically, subpopulations a1 and a6 were enriched with up-regulated signature genes, whereas subpopulation a3 was enriched in down-regulated signature genes (*SI Appendix*, Fig. S5*B*). Therefore, we categorized a1 and a6 as the “AD-up-regulated” subpopulations and a3 as the “AD-down-regulated” subpopulation (Fig. 4*B*).

The transcriptome profile of the AD-down-regulated astrocyte subpopulation was characterized by enriched expression of genes associated with neurotransmitter metabolism, including *SLC1A2* and *GLUL* (Fig. 4 *C* and *D* and *SI Appendix*, Fig. S5*C* and Table S5). Indeed, impaired recycling of neurotransmitters, especially glutamate, results in excitotoxicity and leads to neuronal death (6). The AD-up-regulated subpopulations exhibited enriched expression of stress response-associated genes including *CRYAB* (a heat shock protein), *GFAP* (a reactive astrocyte marker), and *LINGO1* (a negative regulator of myelination) (*SI Appendix*, Fig. S5 *D* and *E*). Moreover, the expression of *HMGB1*, an alarmin that conveys injury signals to surrounding cells, was higher in the AD-up-regulated subpopulations than the AD-down-regulated subpopulation (*SI Appendix*, Fig. S5*D*) (20). Thus, our results show that in AD, (*i*) three specific astrocyte subpopulations contribute to the transcriptomic changes in astrocytes, (*ii*) the subpopulation of homeostatic astrocytes (a3), which are crucial for neurotransmitter recycling, is reduced, and (*iii*) alarmin-expressing astrocytes are induced (a1 and a6). These findings suggest that astrocytic dysfunction in AD contributes to the dysregulation of neurotransmitter recycling and exaggerated alarmin response.

Regarding oligodendrocytes, subcluster analysis identified nine subpopulations in NC and AD brains (Fig. 4*E*). Among these, only four subpopulations—o1, o2, o3, and o5—were enriched with genes that were differentially expressed in oligodendrocytes in AD (Fig. 4*F* and *SI Appendix*, Fig. S5 *F* and *G*). Pathway analysis revealed that the AD-down-regulated oligodendrocyte subpopulations o3 and o5 exhibited enriched expression of genes associated with myelination, including *MAG*, *MOBP*, and *OPALIN* (Fig. 4 *G* and *H* and *SI Appendix*, Fig. S5*H* and Table S5). Along the oligodendroglial lineage, myelinated mature oligodendrocytes exhibit enriched expression of *MAG*, *MOBP*, and *OPALIN* (21). Therefore, we concluded that the o3 and o5 subpopulations were mature myelinated oligodendrocytes. Notably, the transcriptome profiles of the AD-up-regulated oligodendrocyte subpopulations o1 and o2 were characterized by enriched expression of *HSPA1A* (a heat shock protein), *NEAT1* (a nuclear noncoding RNA), and *PDE1A* (which encodes phosphodiesterase 1A) (*SI Appendix*, Fig. S5 *I* and *J*); this expression pattern resembles that of remyelinating oligodendrocytes in patients with multiple sclerosis (22). Thus, our results show that in AD, there is a decrease in the number of mature myelinated oligodendrocytes and an increase in the number of remyelinating oligodendrocytes.

Finally, subcluster analysis of microglia identified 13 subpopulations; only 3 of them—m1, m6, and m7—contributed to the transcriptomic changes in microglia in AD (*SI Appendix*, Fig. S6 *A* and *B* and Table S5). Notably, compared to the NC samples, the AD samples exhibited a smaller proportion of the m6 subpopulation, which expresses genes important for synaptic pruning (i.e., *C1QA*, *C1QB*, and *C1QC*, which encode complement component 1q) and cytokine response (i.e., *IL4R* and *IL1RAP*) (*SI Appendix*, Fig. S6 *C* and *D*). Given that aberrant complement signaling mediates nonselective synaptic pruning via phagocytosis (23), our results suggest that the loss of this typical microglial subpopulation can contribute to the imbalanced complement signaling and synaptic pruning in AD.

### The Induction of Angiogenic Endothelial Cells Contributes to Immune Dysregulation in AD.

**Fig. 5.**
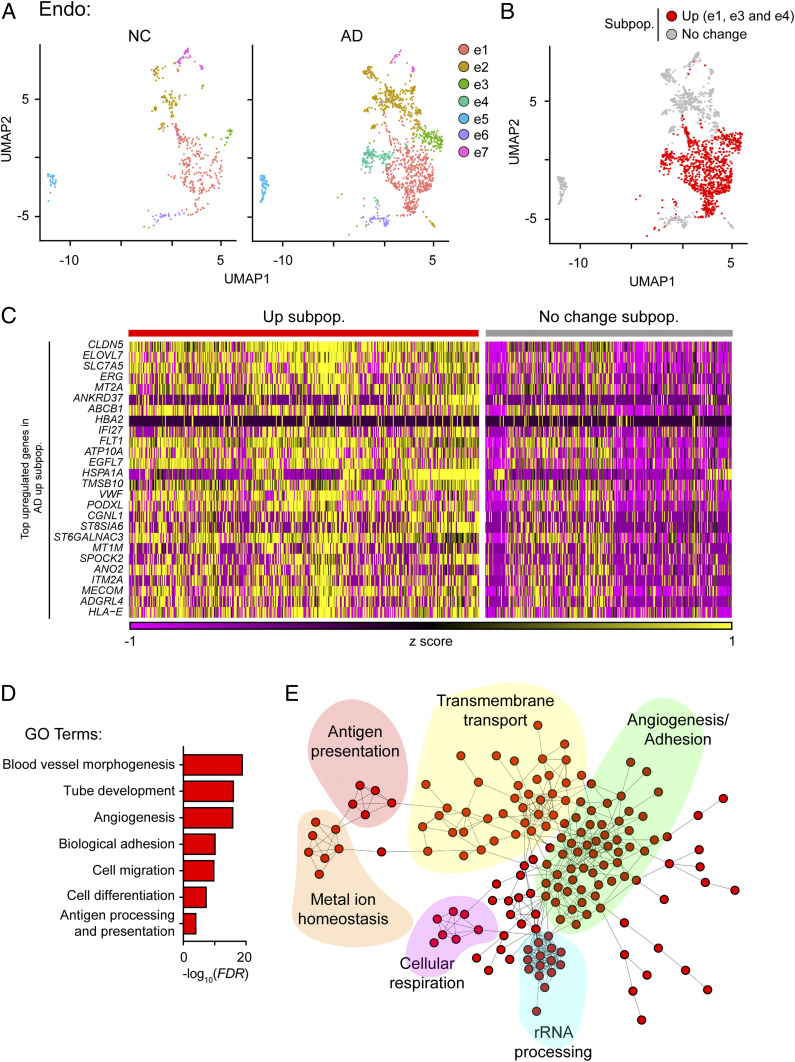
An endothelial cell subpopulation associated with enhanced angiogenesis and antigen presentation is increased in AD. (*A*–*C*) Transcriptomically unique subpopulations of endothelial cells were present in AD samples. (*A*) UMAP plots showing the distributions of endothelial subpopulations (e1–e7) in AD and NC prefrontal cortical samples. (*B*) Distribution of AD-associated endothelial subpopulations. Red: AD-up-regulated subpopulations (i.e., e1, e3, and e4). (*C*) Expression levels of the top enriched genes in the AD-up-regulated subpopulations at the single-cell level (adjusted *P* < 0.1, log_2_ fold change ≥ 0.1). Up subpop.: up-regulated subpopulation. (*D* and *E*) The activated endothelial cells in AD were associated with angiogenesis and antigen presentation. GO analysis of the up-regulated genes in AD-up-regulated endothelial subpopulations (*D*), and STRING analysis (*E*) showing that the signature genes of activated endothelial cells form a protein–protein interaction network associated with six different functional pathways. See also *SI Appendix*, Fig. S7 and Table S5.

Our results demonstrate that the transcriptomic changes of endothelial cells in AD are associated with angiogenesis and immune response (Fig. 2*E*). Our subcluster analysis further revealed that only three of the seven identified endothelial cell subpopulations—e1, e3, and e4—contributed to the transcriptomic changes observed in endothelial cells in AD (Fig. 5*A* and *SI Appendix*, Fig. S7 *A* and *B* and Table S5). Of note, all three subpopulations were classified as AD-up-regulated subpopulations (Fig. 5*B* and *SI Appendix*, Fig. S7*C*). Transcriptome profiling showed that genes associated with angiogenesis (i.e., *CLDN5*, *ERG*, *FLT1*, and *VWF*) and antigen presentation—especially MHC-I (major histocompatibility complex class I) machinery (i.e., *HLA-E*)—were enriched in these three AD-up-regulated subpopulations (Fig. 5*C* and *SI Appendix*, Table S5). Pathway analysis and protein–protein interaction network analysis further revealed that the signature genes of these AD-up-regulated subpopulations form an interaction network associated with six major biological functions: angiogenesis/adhesion, transmembrane transport, antigen presentation, metal ion homeostasis, cellular respiration, and rRNA processing (Fig. 5 *D* and *E*).

We subsequently investigated the potential mechanisms by which angiogenic endothelial cells are induced in AD by performing ingenuity pathway analysis using the signature genes in the previously identified AD-up-regulated endothelial subpopulations. The results revealed that several regulators of signature genes in the AD-up-regulated subpopulations, including IFNG, IRF7, and TCR, were associated with inflammatory response (*SI Appendix*, Fig. S7*D*). This suggests that the induction of angiogenic endothelial cells is regulated by a proinflammatory response. Moreover, analysis of bulk microarray data from mouse models of amyloid-beta deposition or Tau hyperphosphorylation also revealed transcriptome reprogramming similar to that observed in endothelial cells in patients with AD (*SI Appendix*, Fig. S7*E*), suggesting that the activation of endothelial cells in neurodegeneration is conserved between humans and mice.

## Discussion

The identification of precise molecular and cellular targets for AD therapeutic development requires a comprehensive understanding of the cell type-specific responses and cellular heterogeneity in AD. Accordingly, our single-nucleus transcriptome analysis identified the four major molecular pathways that are dysregulated in specific neural cell types in patients with AD: impaired synaptic signaling in astrocytes and neurons, reduced myelination in oligodendrocytes, aberrant immune activation in microglia and endothelial cells, and enhanced angiogenesis in endothelial cells. Moreover, we showed that the dysregulation of these pathways in AD is due to changes in the subpopulation heterogeneity of respective cell types, including the reduced proportions of neuroprotective astrocytes and oligodendrocytes as well as the increased proportions of endothelial cells, which are associated with enhanced angiogenesis and antigen presentation. Together, our single-nucleus transcriptomic profiling highlights the potential roles of dysregulated angiogenesis in endothelial cells of AD patients.

The results of our single-nucleus transcriptomic profiling of AD brains are a useful resource for understanding the cellular dysregulation along AD progression. For example, we showed that the dysregulated pathways in neurons and oligodendrocytes are associated with synaptic signaling and myelination, respectively; these findings are similar to those in previous studies despite variations in study cohorts, brain regions, sample preparation, and sequencing protocols (12, 13, 23). In addition to these consistent changes, our findings provide insights into the cellular changes that occur in AD. For example, we found that the dysregulated pathways in endothelial cells are associated with angiogenesis and antigen presentation. However, such changes were not observed in previous snRNA-seq studies (12, 13), possibly due to a lower proportion of endothelial nuclei being sampled. Therefore, the addition of our dataset to the existing snRNA-seq datasets of AD brains enhances the comprehensiveness of cell type-specific transcriptomic changes in AD, especially in endothelial cells.

In addition to identifying dysregulated pathways involved in AD pathogenesis, our molecular characterization of the human AD brain supports the prevailing hypotheses based on studies of transgenic mouse models of AD. For example, previous studies using transgenic mouse models of AD have identified various mechanisms that lead to synaptic impairment in AD, including the accumulation of soluble amyloid-beta, Tau hyperphosphorylation, loss of VGF signaling, and glial state dysregulation (e.g., the induction of disease-associated microglia and disease-associated astrocytes) (9, 23–30). For astrocytes in particular, dysregulated communication with neurons can impair synaptic plasticity (31, 32). Indeed, we showed that the AD brain contains a reduced proportion of neuroprotective astrocytes, which are associated with glutamate recycling and synaptic signaling. This could lead to synaptic loss due to excitotoxicity (6, 33, 34) and reduced synaptogenesis due to the destabilization of nascent synapses (16, 17), which would result in a net loss of synapses during disease progression. Therefore, our findings suggest that the dysregulated pathways in astrocytes could contribute to synaptic impairment in AD.

Similarly, the loss of myelinated oligodendrocyte subpopulations suggests the disruption of oligodendrocyte maintenance in AD. Indeed, aging-related myelin loss is associated with cognitive impairment in AD (35). In animal models, demyelination can be triggered by the accumulation of amyloid-beta peptides, phosphorylated Tau, or proinflammatory cytokines (35–37). Consistent with recent findings (12, 13), the molecular changes observed in our study were associated with the demyelination process in AD. Interestingly, our results also show that oligodendrocytes adopt a remyelinating state in AD, suggesting a potential cell-intrinsic recovery mechanism. While proper remyelination can restore the impaired saltatory conduction in neurons (38), it remains unclear why remyelinating oligodendrocytes fail to overcome the demyelination observed in AD.

Our transcriptome profiling revealed that endothelial cells adopt an angiogenic state characterized by enhanced expression of genes involved in angiogenesis and antigen presentation. Endothelial cells are the primary component of the neurovascular system, which maintains blood–brain barrier integrity. Both patients with AD and AD animal models exhibit neurovascular system impairment including abnormalities in the number, diameter, and density of blood vessels, which lead to decreased brain perfusion and blood–brain barrier disruption (10, 39, 40). Compromised blood–brain barrier integrity permits the direct entry of neurotoxic thrombin and plasmin, leading to synapse loss (41, 42). Although it remains unclear how the dysregulation of endothelial cells leads to blood–brain barrier abnormalities in AD, our findings show that in AD, endothelial cells adopt an angiogenic state characterized by increased expression of angiogenic factors and receptors including *ERG*, *FLT1*, and *VWF*. Notably, increased expression of angiogenic genes in endothelial cells is associated with the blood vessel abnormalities observed in mouse models of neurodegeneration (40). Therefore, our study provides evidence of a potential link between endothelial angiogenesis and blood–brain barrier abnormalities in patients with AD.

Our findings also suggest a role of endothelial cells in antigen presentation, specifically that by MHC-I, in AD. MHC-I mostly presents endogenous proteins that originate in the cytoplasm, making it essential in the response to viral infection (43). Upon viral infection, endothelial MHC-I presents endogenous viral products and facilitates CD8^+^ T cell activation (44–46). Interestingly, several recent studies suggest that AD is associated with viral infection (47, 48). Therefore, the induction of MHC-I in endothelial cells in AD might reflect response to viral infection. Another possible role of this MHC-I induction is the triggering of CD8^+^ T cell activation and subsequent clonal expansion. Indeed, increased clonal expansion and CD8^+^ T cell infiltration in the brain are observed both in AD patients and during aging (48, 49). Once activated CD8^+^ T cells have infiltrated the brain, they can release IFN-γ to stimulate excessive microglial synaptic pruning or inhibit adult neural stem cell proliferation (44, 49), impairing neuronal homeostasis. Therefore, it is of great interest to investigate how MHC-I induction in endothelial cells contributes to AD pathogenesis.

In summary, our transcriptome profiling results constitute a useful resource for understanding the pathological roles of endothelial and glial cells in AD. Furthermore, our data can aid the identification of endothelial- and glial-specific molecular targets for the therapeutic restoration of neural homeostasis and the amelioration of the pathological progression of this devastating disease.

## Methods

### Selection Criteria for Brain Tissue Collection from AD Patients.

We obtained postmortem prefrontal cortex tissue samples (from the BA6, BA8, and BA9 domains) from patients with AD and NC subjects from the South West Dementia Brain Bank (SWDBB). The clinical diagnosis of AD was based on the Diagnostic and Statistical Manual of Mental Disorders-5 criteria for AD (*SI Appendix*, Table S1). For initial sample selection from the SWDBB, we excluded subjects with other neurodegenerative diseases, vascular diseases, an intoxicated state, infection, prions, inflammatory diseases, structural brain disorders, metabolic/nutritional diseases, trauma, delirium, genetic disorders (e.g., Down syndrome), or other systemic diseases (*SI Appendix*, Table S1). We categorized samples according to Braak stage (i.e., AD: ≥4 and NC: ≤2). For snRNA-seq library preparation, we subsequently selected subjects according to age and postmortem delay and ensured a balanced sex ratio. Thus, we included 21 subjects (mean age: AD, 74.6 y; NC, 85.4 y) including 8 males and 4 females with AD as well as 6 male and 3 female NC subjects. We did not intentionally include/exclude samples according to *APOE* genotype prior to snRNA-seq library preparation but confirmed *APOE* genotypes by TaqMan assay with a probe from Thermo Scientific (assay ID: C_3084793_20 and C_904973_10) following the manufacturer’s instructions.

### Isolation of Nuclei from the Brain Tissues of AD Patients.

We isolated nuclei from frozen prefrontal cortex samples as previously described with minor modifications (50). Briefly, we placed frozen cortical tissues directly into a prechilled Dounce homogenizer with ice-cold homogenization buffer (0.25 M sucrose, 25 mM KCl, 5 mM MgCl_2_, 20 mM tricine-KOH [pH 7.8], 1 mM dithiothreitol, 0.15 mM spermine, 0.5 mM spermidine, protease inhibitors, 5 μg/mL actinomycin, 0.32% Nonidet P-40, and 0.04% bovine serum albumin). After 25 strokes with a loose pestle, we mixed the homogenate 1:1 in OptiPrep and centrifuged the solution at 10,000 × *g* for 20 min at 4 °C. We subsequently collected the separated nuclei, which were pelleted at the bottom of the centrifuge tube, washed them once to remove the OptiPrep, and resuspended them in Dulbecco's modified Eagle medium/F12 supplemented with 10% fetal bovine serum. We diluted the nuclei to 400 nuclei per microliter, ensuring this dilution by counting with a hemocytometer. We also assessed the purity of the single-nucleus suspensions by flow cytometry; this protocol routinely yielded high-purity, single-nucleus suspensions (i.e., >95% DAPI^+^ nuclei in all samples). All buffers and gradient solutions for nuclei extraction contained 60 U/mL RNAsin (Promega).

### snRNA-seq Library Preparation.

We generated snRNA-seq libraries using the Chromium Single Cell 3′ Library Kit v3 (1000078; 10× Genomics) according to the manufacturer’s instructions. For snRNA-seq library construction, we used 40-μL diluted nucleus suspension (400 nuclei per µL) mixed with reverse-transcription reagent mix and loaded the sample into a chip for single-cell encapsulation. We then immediately incubated the encapsulated nuclei on a thermocycler to enable reverse transcription of the RNA and generate barcoded cDNA. We used this cDNA for library construction according to the manufacturer’s instructions. The concentrations of the final libraries were determined by Qubit (Thermo Fisher Scientific), and the fragment lengths were determined by a Fragment Analyzer (Advanced Analytical Technologies). We subjected libraries to paired-end sequencing on a NovaSeq 6000 system according to the manufacturer’s instructions (Novogene), and at least 200 GB of raw data were obtained per library.

### snRNA-seq Analysis.

#### Preprocessing and quality control.

Given that snRNA-seq libraries capture reads from both unspliced pre-messenger RNAs (mRNAs) and mature mRNAs, we first generated a pre-mRNA reference genome according to the instructions provided by 10× Genomics. We subsequently aligned the demultiplexed FASTQ files from Novogene to the GRCh38 pre-mRNA reference genome using Cell Ranger (version 3.0.1) with the default settings (51). After performing the alignment, we used the default quality control settings in Cell Ranger as an initial quality control step. This initial quality control process retained only barcodes with unique molecular identifier counts within the top 10% of the 99th percentile of unique molecular identifier values among all barcodes and sorted them into cell-associated matrixes; the remaining barcodes were treated as background barcodes and were excluded. We subsequently used these cell-associated matrixes as input for the second round of quality control and downstream analysis in Seurat (version 3.0) (52).

For the second round of quality control, we controlled for the distributions of the numbers of genes, numbers of unique molecular identifier counts, and percentages of mitochondrial genes for each sample in Seurat. To exclude potential dead cells and cell debris from the dataset, we filtered out nuclei with ≤200 genes, ≥20,000 unique molecular identifiers, or ≥20% mitochondrial genes as described in several previous snRNA-seq studies (12, 13, 22, 53). The final filtered matrix contained 169,496 nuclei and 29,171 genes. During sample preparation, we targeted ∼16,000 nuclei per sample and found that while the number of nuclei that passed quality control varied among samples, the variation was independent of age, sex, postmortem delay, and pathology, and there was no batch effect (*SI Appendix*, Table S1). Although the postmortem delay varied among samples, we also found that the numbers of genes and detected transcripts were independent of postmortem delay variation (*SI Appendix*, Fig. S1*A*).

#### Cell-type identification by dimensionality reduction.

For integrative analysis, we followed the workflow described in the Seurat guided analysis. We first log-normalized the filtered matrixes and identified highly variable features for each sample using the *FindVariableFeatures* function with the parameters *selection.method* = *vst*, and *nfeatures = 1000*. To integrate all 21 samples, we identified features for anchoring the samples using the *FindIntegrationAnchors* function with the parameter *dims = 1:20* and used the identified anchors to integrate the dataset using the *IntegrateData* function with the parameter *dims = 1:20*. We subsequently scaled the integrated matrix and performed linear dimensional reduction using the *RunPCA* function with the parameter *npcs = 50*. We visualized the *P* value distribution of each principal component using the *JackStrawPlot* function and opted to use the first 20 principal components for graph-based clustering.

We performed *K*-nearest neighbor clustering using the *FindClusters* function with the parameter *resolution = 1* and UMAP clustering using the *RunUMAP* function with the parameter *dims = 1:20*, which initially yielded 43 cell clusters. We identified the DEGs in each cell cluster by the Wilcoxon rank-sum test using the *FindAllMarkers* function with the parameters *logfc.threshold = 0.25* and *test.use = wilcox*. We then assigned a cell-type identity to each cell cluster according to the expression of known cell-type markers and identified additional cell type-specific marker genes by the Wilcoxon rank-sum test using the *FindAllMarkers* function with the parameters *logfc.threshold = 0.25* and *test.use = wilcox*. For cell-type markers, the level of statistical significance was set at an adjusted *P* value <0.1. To further confirm the cell-type specificity of these markers, we compared our data with those of Jäkel et al. (22), which confirmed the expression patterns of these markers in the different cell types in our study.

#### Examination of cell type-specific transcriptomic changes.

To examine the cell type-specific transcriptomic changes in AD, we stratified samples according to our initial classification of AD and NC samples (based on Braak stage) and compared the transcriptome profiles of individual cell types between AD and NC samples by the Wilcoxon rank-sum test using the *FindMarkers* function with the parameters *logfc.threshold = 0* and *test.use = wilcox*. The level of statistical significance for cell type-specific transcriptomic changes was set at an adjusted *P* < 0.1 and a log_2_ fold change ≥0.1 or ≤ −0.1.

#### Subcluster analysis.

For subcluster analysis, we first isolated individual cell types from the original Seurat dataset using the *Subset* function. We subsequently reclustered each cell type using an approach similar to that used for our initial cell type clustering. We performed *K*-nearest neighbor clustering using the *FindClusters* function with the parameters *resolution = 0.2*, *0.2*, *0.3*, and *0.2* for astrocytes, endothelial cells, microglia, and oligodendrocytes, respectively, as well as UMAP clustering using the *RunUMAP* function with the parameter *dims = 1:20*. To perform unbiased identification of the subpopulations of cells that contribute to AD-associated transcriptomic changes, we calculated the enrichment scores of AD-associated DEGs in each subpopulation by averaging the *z*-scores of all AD-associated DEGs. A subpopulation with an enrichment score > 0.5 was designated as an AD-associated subpopulation. We identified the transcriptomic signatures of AD-associated subpopulations by comparing up- and down-regulated subpopulations using the Wilcoxon rank-sum test with the *FindMarkers* function and the parameters *logfc.threshold = 0* and *test.use = wilcox*. The level of statistical significance was set at an adjusted *P* < 0.1 and a log_2_ fold change ≥ 0.1 or ≤ −0.1.

### Data Validation by Comparison with Previous Studies.

For data validation, we obtained datasets from published studies and compared them with our findings. We obtained DEG analysis results from a recent snRNA-seq study (12). We selected a list of cell type-specific DEGs between healthy and diseased samples with an adjusted *P* < 0.01 (two-sided Wilcoxon rank-sum test) and log_2_ fold change ≥ 0.25 or ≤ −0.25. However, the proportion of endothelial cells in the previous dataset was low, rendering it unsuitable for validating our findings in endothelial cells.

We also obtained the dataset from a study by Narayanan et al. (Gene Expression Omnibus [GEO] accession no. GSE33000), who performed bulk transcriptome microarray analysis of prefrontal cortical tissues in a large cohort (AD: *n* = 310; NC: *n* = 157) (18). We first filtered the samples according to disease status and kept only “Alzheimer’s disease” and “non-demented” samples for subsequent analysis. We removed genes that failed to be mapped to Entrez gene IDs. For genes mapped by multiple probes, we used the median value. To scale the variance, we performed log_2_ transformation and quantile normalization using the R *limma* package (54). For differential expression analysis, we fitted the gene expression profiles by linear regression, adjusting for age and sex. We used the empirical Bayes method provided in *limma* to calculate *t*-statistics and log-fold changes in differential expression. We adjusted the *P* values using the Benjamini–Hochberg procedure. We also used the same pipeline to process the microarray dataset of AD temporal cortical samples from Webster et al. (GEO accession no. GSE15222) (19).

We obtained transcriptome data for mouse models of amyloid-beta deposition and Tau hyperphosphorylation from MOUSEAC (55).

### Data Visualization.

We visualized the data using Morpheus, Seurat’s *DoHeatmap* or *DotPlot* function, or Cytoscape (version 3.7.0) where appropriate. DEGs were functionally annotated according to Gene Ontology (GO) analysis (geneontology.org), ingenuity pathway analysis (QIAGEN), and STRING analysis (https://string-db.org).

## Supplementary Material

Supplementary File

Supplementary File

Supplementary File

Supplementary File

Supplementary File

Supplementary File

## Data Availability

Anonymized snRNA-seq sequencing data have been deposited in GEO (accession no. GSE157827). All study data are included in the article and supporting information.

## References

[1] Reitz C., Brayne C., Mayeux R., Epidemiology of Alzheimer disease. Nat. Rev. Neurol. 7, 137–152 (2011). 21304480 10.1038/nrneurol.2011.2 PMC3339565

[2] Fu W. Y., Wang X., Ip N. Y., Targeting neuroinflammation as a therapeutic strategy for Alzheimer’s disease: Mechanisms, drug candidates, and new opportunities. ACS Chem. Neurosci. 10, 872–879 (2019). 30221933 10.1021/acschemneuro.8b00402

[3] Snyder E. M.., Regulation of NMDA receptor trafficking by amyloid-β. Nat. Neurosci. 8, 1051–1058 (2005). 16025111 10.1038/nn1503

[4] Chen Y., Fu A. K. Y., Ip N. Y., Synaptic dysfunction in Alzheimer’s disease: Mechanisms and therapeutic strategies. Pharmacol. Ther. 195, 186–198 (2019). 30439458 10.1016/j.pharmthera.2018.11.006

[5] Scheff S. W., Price D. A., Schmitt F. A., Mufson E. J., Hippocampal synaptic loss in early Alzheimer’s disease and mild cognitive impairment. Neurobiol. Aging 27, 1372–1384 (2006). 16289476 10.1016/j.neurobiolaging.2005.09.012

[6] Hynd M. R., Scott H. L., Dodd P. R., Glutamate-mediated excitotoxicity and neurodegeneration in Alzheimer’s disease. Neurochem. Int. 45, 583–595 (2004). 15234100 10.1016/j.neuint.2004.03.007

[7] Parkhurst C. N.., Microglia promote learning-dependent synapse formation through brain-derived neurotrophic factor. Cell 155, 1596–1609 (2013). 24360280 10.1016/j.cell.2013.11.030 PMC4033691

[8] Christopherson K. S.., Thrombospondins are astrocyte-secreted proteins that promote CNS synaptogenesis. Cell 120, 421–433 (2005). 15707899 10.1016/j.cell.2004.12.020

[9] Liddelow S. A.., Neurotoxic reactive astrocytes are induced by activated microglia. Nature 541, 481–487 (2017). 28099414 10.1038/nature21029 PMC5404890

[10] Zlokovic B. V., Neurovascular pathways to neurodegeneration in Alzheimer’s disease and other disorders. Nat. Rev. Neurosci. 12, 723–738 (2011). 22048062 10.1038/nrn3114 PMC4036520

[11] Zhao Z., Nelson A. R., Betsholtz C., Zlokovic B. V., Establishment and dysfunction of the blood-brain barrier. Cell 163, 1064–1078 (2015). 26590417 10.1016/j.cell.2015.10.067 PMC4655822

[12] Mathys H.., Single-cell transcriptomic analysis of Alzheimer’s disease. Nature 570, 332–337 (2019). 31042697 10.1038/s41586-019-1195-2 PMC6865822

[13] Grubman A.., A single-cell atlas of entorhinal cortex from individuals with Alzheimer’s disease reveals cell-type-specific gene expression regulation. Nat. Neurosci. 22, 2087–2097 (2019). 31768052 10.1038/s41593-019-0539-4

[14] Wang X.., Deciphering cellular transcriptional alterations in Alzheimer’s disease brains. Mol. Neurodegener. 15, 38 (2020). 32660529 10.1186/s13024-020-00392-6 PMC7359236

[15] Habib N.., Massively parallel single-nucleus RNA-seq with DroNc-seq. Nat. Methods 14, 955–958 (2017). 28846088 10.1038/nmeth.4407 PMC5623139

[16] Stogsdill J. A.., Astrocytic neuroligins control astrocyte morphogenesis and synaptogenesis. Nature 551, 192–197 (2017). 29120426 10.1038/nature24638 PMC5796651

[17] Südhof T. C., Synaptic neurexin complexes: A molecular code for the logic of neural circuits. Cell 171, 745–769 (2017). 29100073 10.1016/j.cell.2017.10.024 PMC5694349

[18] Narayanan M.., Common dysregulation network in the human prefrontal cortex underlies two neurodegenerative diseases. Mol. Syst. Biol. 10, 743 (2014). 25080494 10.15252/msb.20145304 PMC4299500

[19] Webster J. A..; NACC-Neuropathology Group, Genetic control of human brain transcript expression in Alzheimer disease. Am. J. Hum. Genet. 84, 445–458 (2009). 19361613 10.1016/j.ajhg.2009.03.011 PMC2667989

[20] Harris H. E., Andersson U., Pisetsky D. S., HMGB1: A multifunctional alarmin driving autoimmune and inflammatory disease. Nat. Rev. Rheumatol. 8, 195–202 (2012). 22293756 10.1038/nrrheum.2011.222

[21] Marques S.., Oligodendrocyte heterogeneity in the mouse juvenile and adult central nervous system. Science 352, 1326–1329 (2016). 27284195 10.1126/science.aaf6463 PMC5221728

[22] Jäkel S.., Altered human oligodendrocyte heterogeneity in multiple sclerosis. Nature 566, 543–547 (2019). 30747918 10.1038/s41586-019-0903-2 PMC6544546

[23] Hong S.., Complement and microglia mediate early synapse loss in Alzheimer mouse models. Science 352, 712–716 (2016). 27033548 10.1126/science.aad8373 PMC5094372

[24] Sheng M., Sabatini B. L., Südhof T. C., Synapses and Alzheimer’s disease. Cold Spring Harb. Perspect. Biol. 4, 10 (2012). 10.1101/cshperspect.a005777 PMC3331702 22491782

[25] Tremblay M. È., Sierra A., Microglia in Health and Disease, (Springer, 2014).

[26] El Gaamouch F.., VGF-derived peptide TLQP-21 modulates microglial function through C3aR1 signaling pathways and reduces neuropathology in 5xFAD mice. Mol. Neurodegener. 15, 4 (2020). 31924226 10.1186/s13024-020-0357-x PMC6954537

[27] Butovsky O.., Identification of a unique TGF-β-dependent molecular and functional signature in microglia. Nat. Neurosci. 17, 131–143 (2014). 24316888 10.1038/nn.3599 PMC4066672

[28] Habib N.., Disease-associated astrocytes in Alzheimer’s disease and aging. Nat. Neurosci. 23, 701–706 (2020). 32341542 10.1038/s41593-020-0624-8 PMC9262034

[29] Keren-Shaul H.., A unique microglia type Associated with restricting development of Alzheimer’s disease. Cell 169, 1276–1290.e17 (2017). 28602351 10.1016/j.cell.2017.05.018

[30] Beckmann N. D.., Multiscale causal networks identify VGF as a key regulator of Alzheimer’s disease. Nat. Commun. 11, 3942 (2020). 32770063 10.1038/s41467-020-17405-z PMC7414858

[31] Hillen A. E. J., Burbach J. P. H., Hol E. M., Cell adhesion and matricellular support by astrocytes of the tripartite synapse. Prog. Neurobiol. 165-167, 66–86 (2018). 29444459 10.1016/j.pneurobio.2018.02.002

[32] Perea G., Navarrete M., Araque A., Tripartite synapses: Astrocytes process and control synaptic information. Trends Neurosci. 32, 421–431 (2009). 19615761 10.1016/j.tins.2009.05.001

[33] Eid T., Tu N., Lee T. S. W., Lai J. C. K., Regulation of astrocyte glutamine synthetase in epilepsy. Neurochem. Int. 63, 670–681 (2013). 23791709 10.1016/j.neuint.2013.06.008 PMC3825815

[34] Petr G. T.., Conditional deletion of the glutamate transporter GLT-1 reveals that astrocytic GLT-1 protects against fatal epilepsy while neuronal GLT-1 contributes significantly to glutamate uptake into synaptosomes. J. Neurosci. 35, 5187–5201 (2015). 25834045 10.1523/JNEUROSCI.4255-14.2015 PMC4380995

[35] Bartzokis G., Age-related myelin breakdown: A developmental model of cognitive decline and Alzheimer’s disease. Neurobiol. Aging 25, 5–18, author reply 49–62 (2004). 14675724 10.1016/j.neurobiolaging.2003.03.001

[36] Anderson J. M.., Abnormally phosphorylated tau is associated with neuronal and axonal loss in experimental autoimmune encephalomyelitis and multiple sclerosis. Brain 131, 1736–1748 (2008). 18567922 10.1093/brain/awn119

[37] Stadelmann C., Wegner C., Brück W., Inflammation, demyelination, and degeneration - recent insights from MS pathology. Biochim. Biophys. Acta 1812, 275–282 (2011). 20637864 10.1016/j.bbadis.2010.07.007

[38] Franklin R. J. M., Ffrench-Constant C., Remyelination in the CNS: From biology to therapy. Nat. Rev. Neurosci. 9, 839–855 (2008). 18931697 10.1038/nrn2480

[39] Sweeney M. D., Sagare A. P., Zlokovic B. V., Blood-brain barrier breakdown in Alzheimer disease and other neurodegenerative disorders. Nat. Rev. Neurol. 14, 133–150 (2018). 29377008 10.1038/nrneurol.2017.188 PMC5829048

[40] Bennett R. E.., Tau induces blood vessel abnormalities and angiogenesis-related gene expression in P301L transgenic mice and human Alzheimer’s disease. Proc. Natl. Acad. Sci. U.S.A. 115, E1289–E1298 (2018). 29358399 10.1073/pnas.1710329115 PMC5819390

[41] Mhatre M.., Thrombin, a mediator of neurotoxicity and memory impairment. Neurobiol. Aging 25, 783–793 (2004). 15165703 10.1016/j.neurobiolaging.2003.07.007

[42] Chen Z. L., Strickland S., Neuronal death in the hippocampus is promoted by plasmin-catalyzed degradation of laminin. Cell 91, 917–925 (1997). 9428515 10.1016/s0092-8674(00)80483-3

[43] Neefjes J., Jongsma M. L. M., Paul P., Bakke O., Towards a systems understanding of MHC class I and MHC class II antigen presentation. Nat. Rev. Immunol. 11, 823–836 (2011). 22076556 10.1038/nri3084

[44] Di Liberto G.., Neurons under T Cell attack coordinate phagocyte-mediated synaptic stripping. Cell 175, 458–471.e19 (2018). 30173917 10.1016/j.cell.2018.07.049

[45] Razakandrainibe R., Pelleau S., Grau G. E., Jambou R., Antigen presentation by endothelial cells: What role in the pathophysiology of malaria? Trends Parasitol. 28, 151–160 (2012). 22365903 10.1016/j.pt.2012.01.004

[46] Pober J. S., Merola J., Liu R., Manes T. D., Antigen presentation by vascular cells. Front. Immunol. 8, 1907 (2017). 29312357 10.3389/fimmu.2017.01907 PMC5744398

[47] Eimer W. A.., Alzheimer’s disease-associated β-Amyloid is rapidly seeded by herpesviridae to protect against brain infection. Neuron 99, 56–63.e3 (2018). 30001512 10.1016/j.neuron.2018.06.030 PMC6075814

[48] Gate D.., Clonally expanded CD8 T cells patrol the cerebrospinal fluid in Alzheimer’s disease. Nature 577, 399–404 (2020). 31915375 10.1038/s41586-019-1895-7 PMC7445078

[49] Dulken B. W.., Single-cell analysis reveals T cell infiltration in old neurogenic niches. Nature 571, 205–210 (2019). 31270459 10.1038/s41586-019-1362-5 PMC7111535

[50] Renthal W.., Characterization of human mosaic Rett syndrome brain tissue by single-nucleus RNA sequencing. Nat. Neurosci. 21, 1670–1679 (2018). 30455458 10.1038/s41593-018-0270-6 PMC6261686

[51] Zheng G. X. Y.., Massively parallel digital transcriptional profiling of single cells. Nat. Commun. 8, 14049 (2017). 28091601 10.1038/ncomms14049 PMC5241818

[52] Butler A., Hoffman P., Smibert P., Papalexi E., Satija R., Integrating single-cell transcriptomic data across different conditions, technologies, and species. Nat. Biotechnol. 36, 411–420 (2018). 29608179 10.1038/nbt.4096 PMC6700744

[53] Zhou Y.., Human and mouse single-nucleus transcriptomics reveal TREM2-dependent and TREM2-independent cellular responses in Alzheimer’s disease. Nat. Med. 26, 131–142 (2020). 31932797 10.1038/s41591-019-0695-9 PMC6980793

[54] Ritchie M. E.., Limma powers differential expression analyses for RNA-sequencing and microarray studies. Nucleic Acids Res. 43, e47 (2015). 25605792 10.1093/nar/gkv007 PMC4402510

[55] Matarin M.., A genome-wide gene-expression analysis and database in transgenic mice during development of amyloid or tau pathology. Cell Rep. 10, 633–644 (2015). 25620700 10.1016/j.celrep.2014.12.041

