# Supplementary material for: Single-nucleus transcriptome analysis reveals dysregulation of angiogenic endothelial cells and neuroprotective glia in Alzheimer’s disease
Source: Proc Natl Acad Sci U S A. 2020 Sep 28;117(41):25800–9. doi: 10.1073/pnas.2008762117 (PMC7568283; doi:10.1073/pnas.2008762117)
Supplement: Supplementary File [file pnas.2008762117.sapp.pdf]

PNAS
www.pnas.org

Supplementary Information for

Single-nucleus transcriptome analysis reveals dysregulation of angiogenic endothelial cells and neuroprotective glia in Alzheimer's disease

Shun-Fat Lau, Han Cao, Amy K.Y. Fu, Nancy Y. Ip[1]

[1] Corresponding author: Nancy Y. Ip

Email: boip@ust.hk

This PDF file includes:

Figures S1 to S7

Other supplementary materials for this manuscript include the following:

Datasets S1 to S5

www.pnas.org/cgi/doi/10.1073/pnas.2008762117

Figure S1. Related to Figure 1.

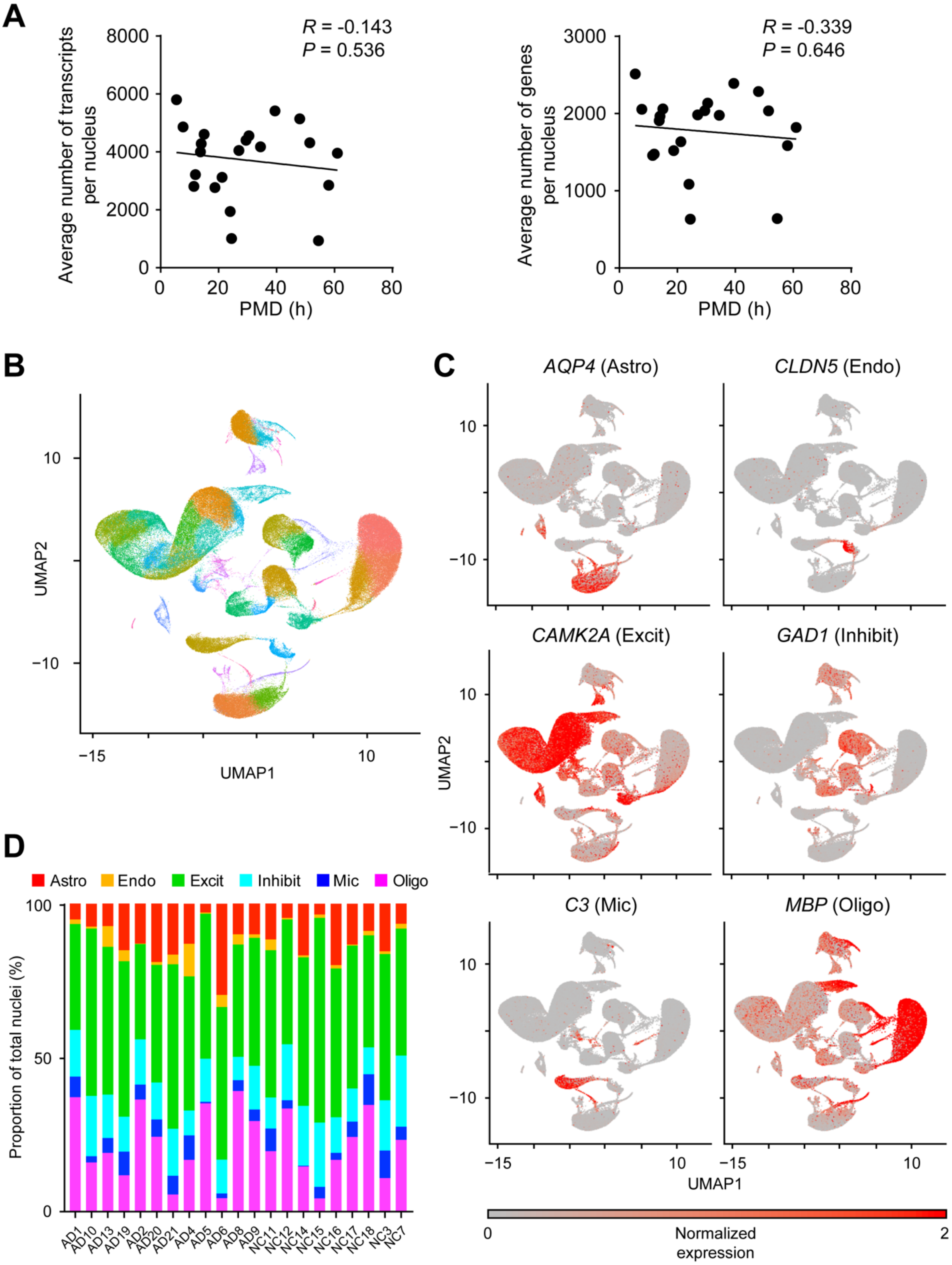

Figure S1. Cell-type heterogeneity in the prefrontal cortex (related to Figure 1)

(A) Correlation between the average number of transcripts (left) and genes (right) per nucleus and postmortem delay (PMD) in all 21 samples. (B) Uniform manifold approximation and projection (UMAP) plot showing the 43 initial cell clusters identified by single-nucleus RNA sequencing. (C) Expression levels of known markers in each cell cluster. Astro: astrocytes, Endo: endothelial cells, Excit: excitatory neurons, Inhibit: inhibitory neurons, Mic: microglia, Oligo: oligodendrocytes. (D) Proportions of the 6 major cell types in each sample.

Figure S2. Related to Figure 2.

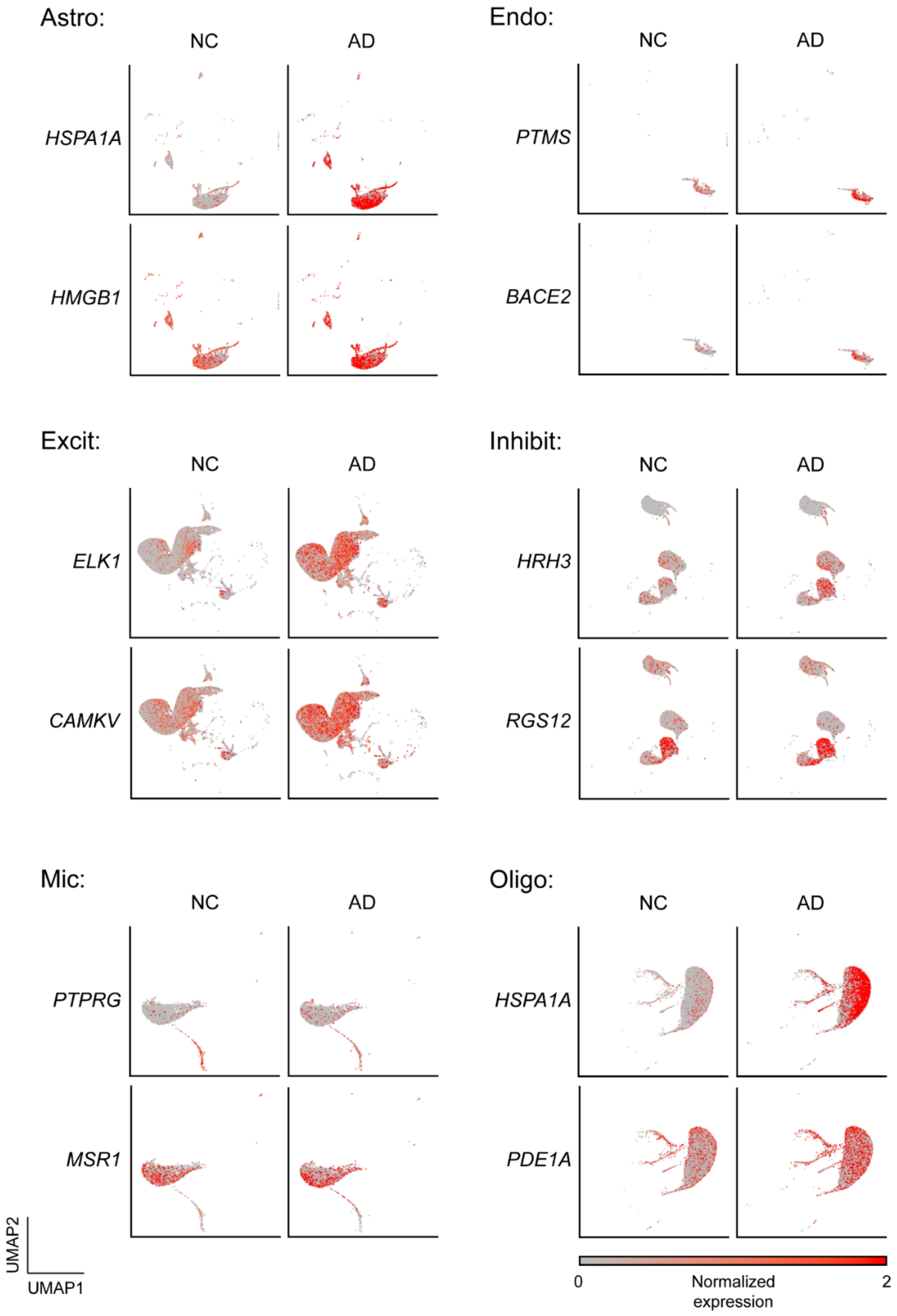

Figure S2. Cell type-specific transcriptomic changes in tissue from patients with Alzheimer's disease (related to Figure 2)

Uniform manifold approximation and projection (UMAP) plots showing the expression levels of the top upregulated genes in each cell type in Alzheimer's disease (AD) and normal control (NC) samples. Astro: astrocytes, Endo: endothelial cells, Excit: excitatory neurons, Inhibit: inhibitory neurons, Mic: microglia, Oligo: oligodendrocytes.

Figure S3. Related to Figure 3.

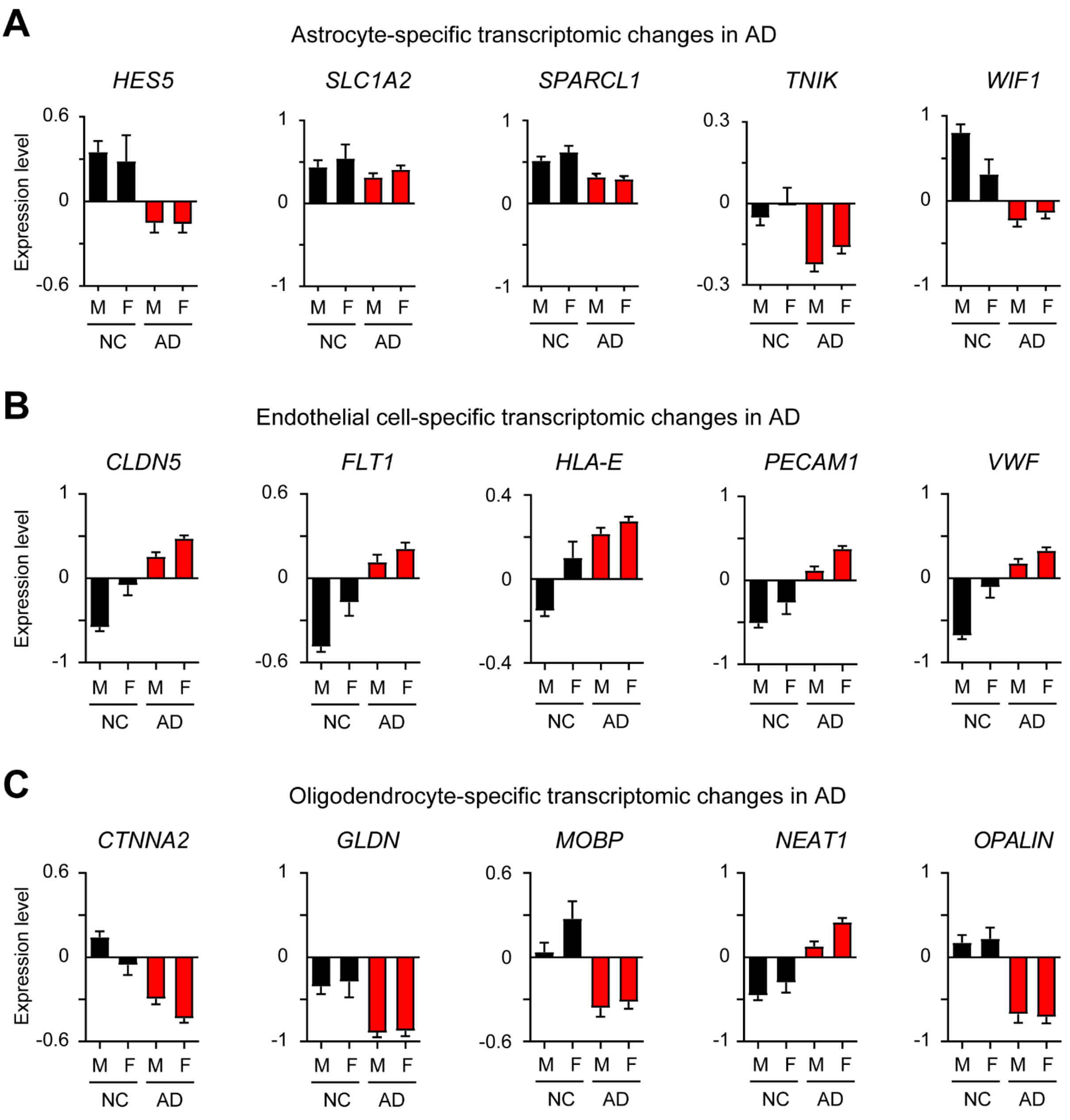

Figure S3. Validation of transcriptomic changes in tissues from patients with Alzheimer's disease based on sex (related to Figure 3)

(A–C) Bar plots showing the expression levels of the top cell type-specific differentially expressed genes in (A) astrocytes, (B) endothelial cells, and (C) oligodendrocytes from male (M) and female (F) patients with Alzheimer's disease (AD) and normal control (NC) subjects. All data are the mean ± SEM.

Figure S4. Related to Figure 2.

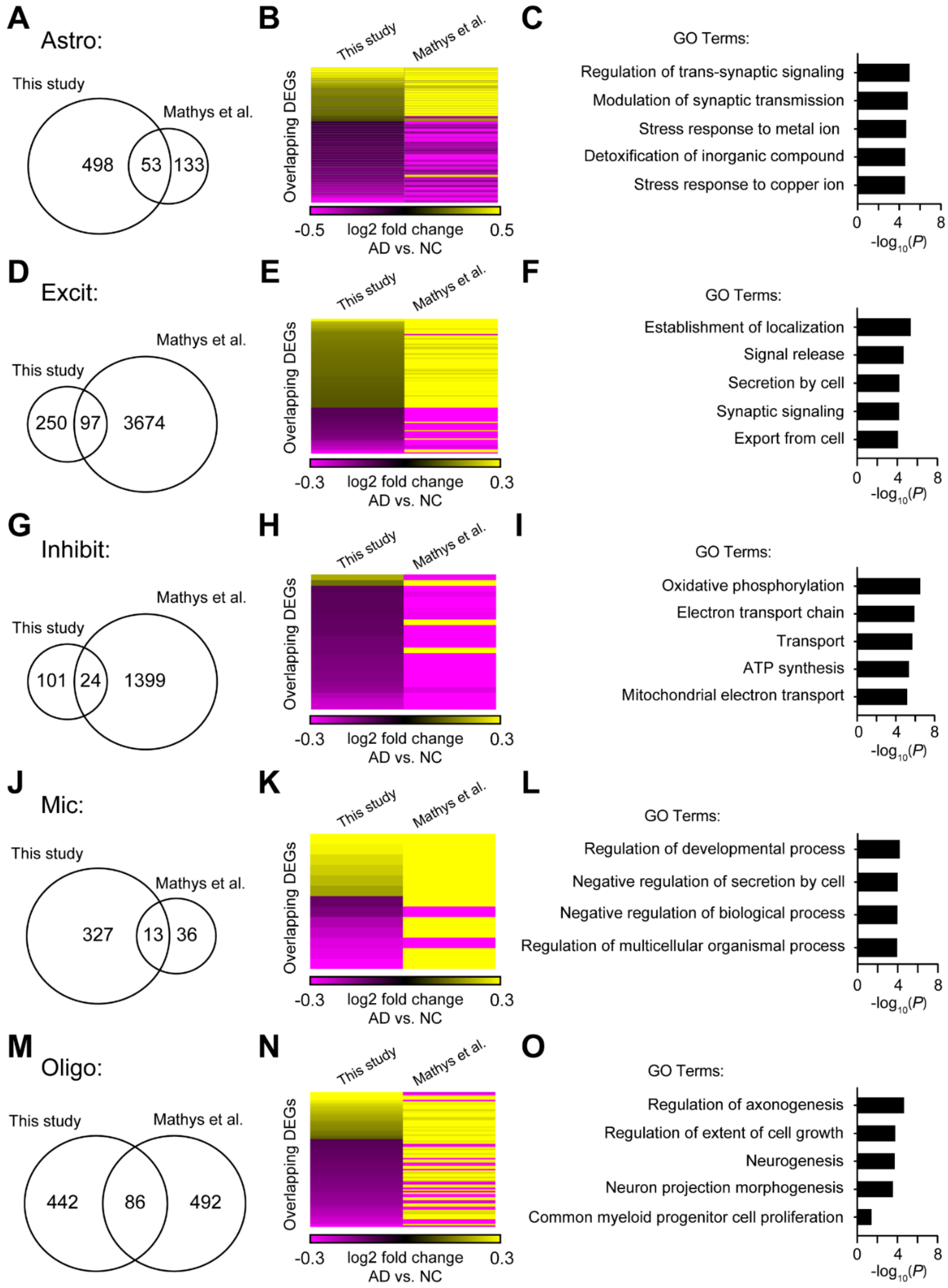

Figure S4. Interstudy comparison of single-nucleus RNA-sequencing analysis of prefrontal cortex samples from patients with Alzheimer's disease (related to Figure 2)

Comparison of the cell type-specific transcriptomic changes in patients with Alzheimer's disease (AD) identified in the present study and those found by Mathys *et al*. Astro: astrocytes, Excit: excitatory neurons, Inhibit: inhibitory neurons, Mic: microglia, and Oligo: oligodendrocytes. (A, D, G, J, M) Venn diagrams comparing the differentially expressed genes (DEGs) specific to astrocytes (A), excitatory neurons (D), inhibitory neurons (G), microglia (J), and oligodendrocytes (M) in the present study with those identified by Mathys *et al*. (B, E, H, K, N) Heatmaps showing the expression changes in the overlapping DEGs in this study and those identified by Mathys *et al*. NC, normal control. (C, F, I, L, O) Bar plots showing the results of Gene Ontology (GO) pathway analysis of the overlapping DEGs.

Figure S5. Related to Figure 4.

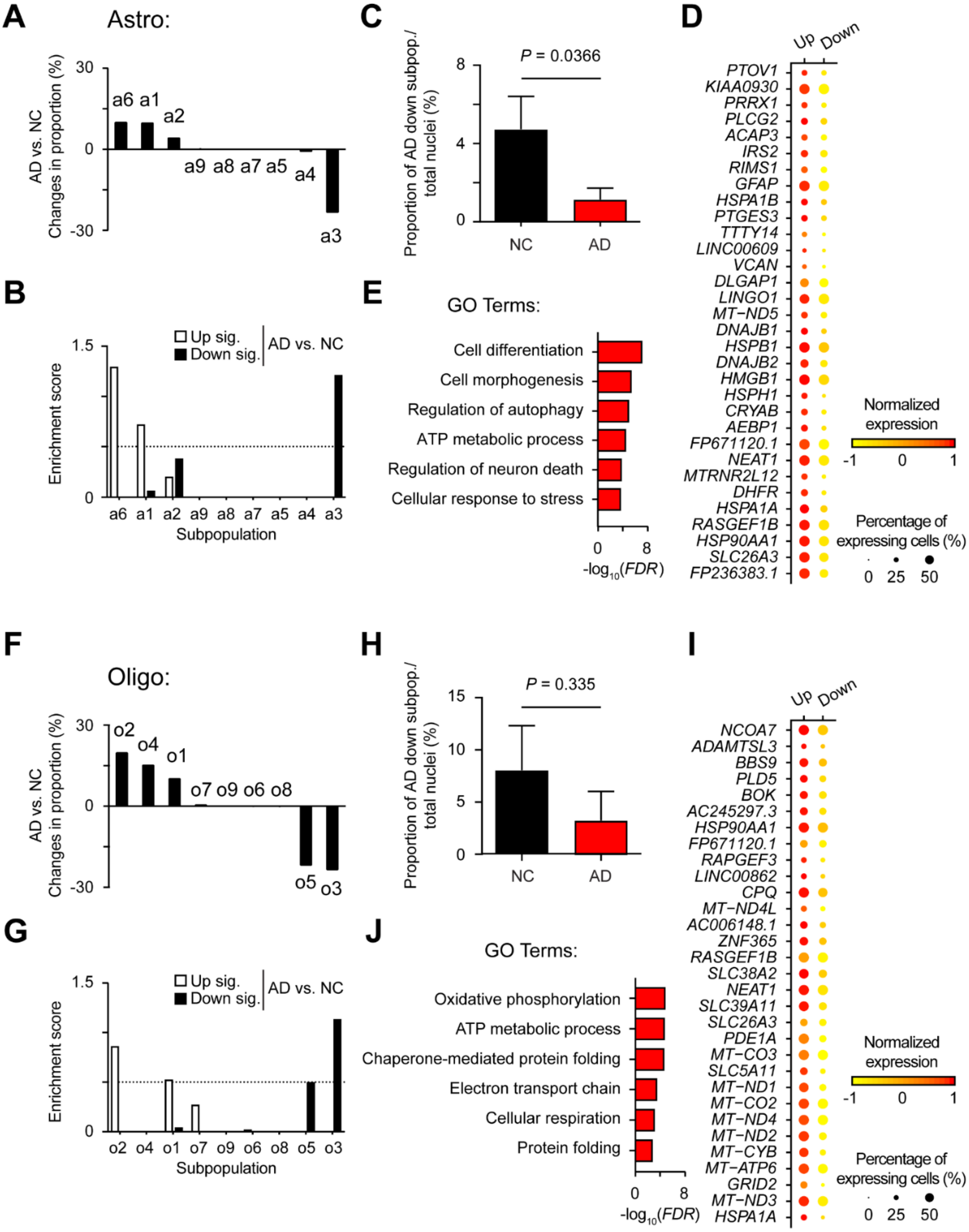

Figure S5. Changes in astrocyte and oligodendrocyte subpopulation heterogeneity in tissues from patients with Alzheimer's disease (related to Figure 4)

(A–J) Induction of stress response-associated astrocytes and oligodendrocytes in tissues from patients with Alzheimer's disease (AD). Astro: astrocytes, Oligo: oligodendrocytes. Changes in the proportions of (A) astrocyte subpopulations (a1–a9) and (F) oligodendrocyte subpopulations (o1–o9) between AD and normal control (NC) samples. (B, G) Only a few glial cell subpopulations contributed to the transcriptomic changes in AD. Bar plots showing the enrichment scores for the AD-associated transcriptomic signature of each cell subpopulation. Up sig.: upregulated signature; Down sig: downregulated signature. The enrichment scores were calculated by averaging the *z*-scores of individual genes in the transcriptomic signature (only positive values are shown). The dotted line indicates the cutoff of *z*-score = 0.5. (C, H) Bar plots showing the proportions of AD-downregulated (C) astrocytes and (H) oligodendrocytes in AD and NC samples. (D, E, I, J) Transcriptome analysis revealed an association between AD-upregulated glial subpopulations and the stress response. (D, I) The top enriched genes in the AD-upregulated glial cell subpopulations. (E, J) Gene Ontology (GO) pathway analysis of the transcriptomic signatures of the AD-upregulated glial cell subpopulations. All data are the mean ± SEM.

Figure S6. Related to Figure 4.

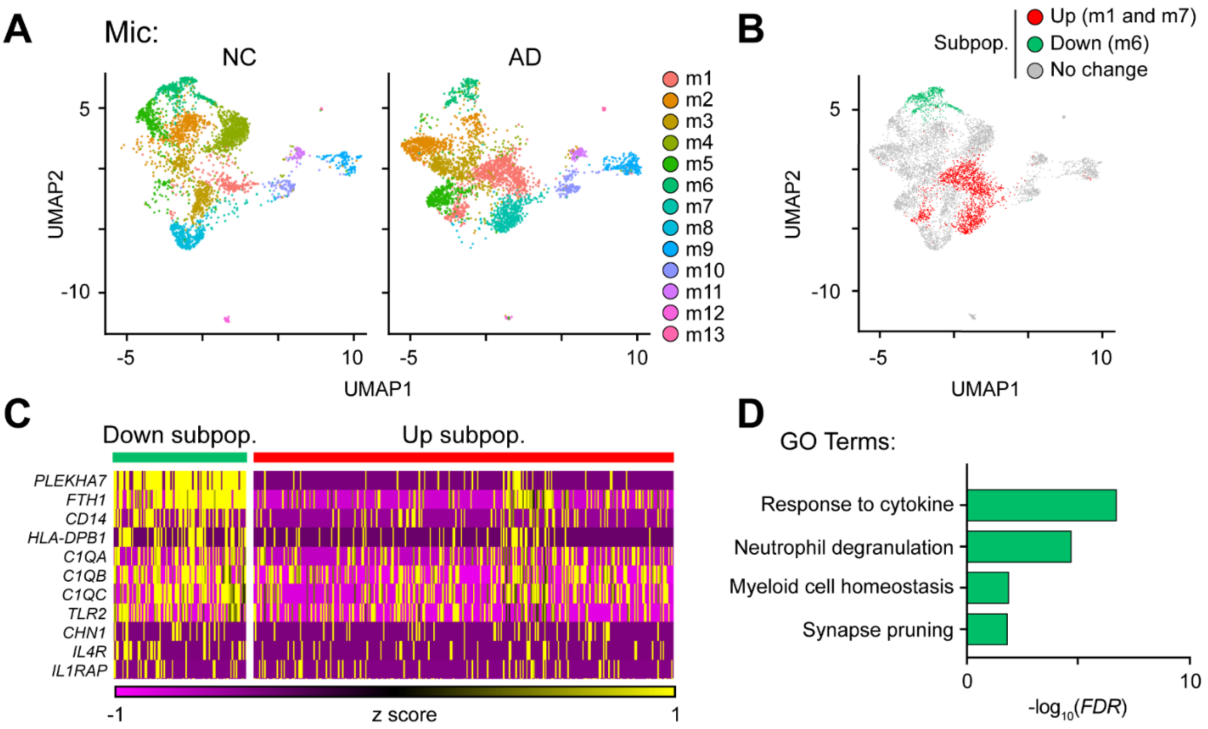

Figure S6. Changes in microglial subpopulation heterogeneity in tissues from patients with Alzheimer's disease (related to Figure 4)

(A–D) Subpopulations of homeostatic microglia (Mic) associated with the cytokine response and synaptic pruning are reduced in tissues from patients with Alzheimer's disease (AD). (A) Uniform manifold approximation and projection (UMAP) plots showing the distribution of microglial subpopulations (m1–m13) in AD and normal control (NC) samples. (B) UMAP plot showing the distribution of the AD-associated microglial subpopulations. Red: AD-upregulated subpopulations (m1 and m7); Green: AD-downregulated subpopulation (m6). (C) Top enriched genes in the AD-downregulated microglial subpopulation (adjusted P < 0.1, $\log_2$ fold change ≥ 0.1). Down subpop.: downregulated subpopulation; Up subpop.: upregulated subpopulation. (D) Gene Ontology (GO) pathway analysis of the transcriptomic signature of the AD-downregulated subpopulation.

Figure S7. Related to Figure 5.

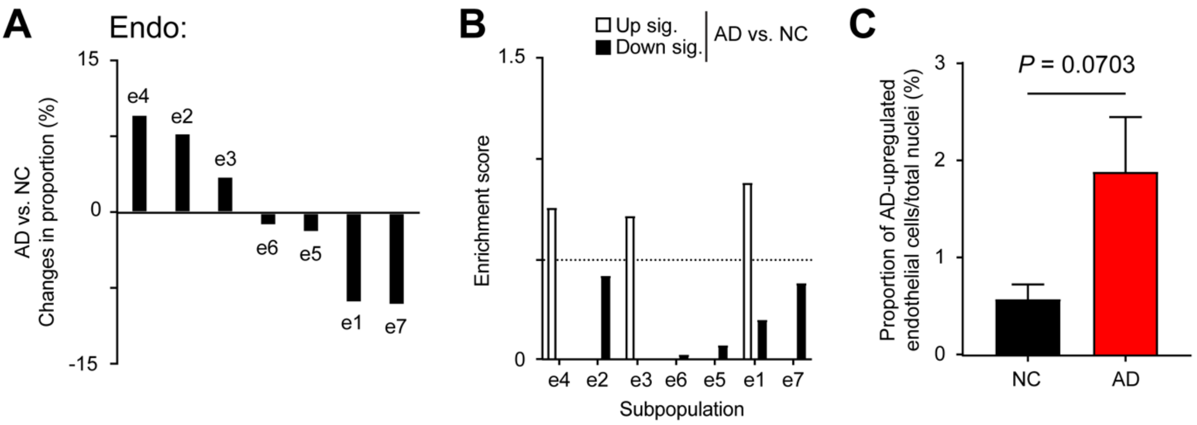

D

Top upstream regulators

Regulator	Type	z score	p
MLXIPL	TF	6.6	9.71E-22
IFNG	Cytokine	6.2	3.05E-22
IFNA2	Cytokine	5.6	1.04E-11
VEGF	Growth factor	5.5	1.5E-17
IRF7	TF	5.1	1.22E-4
TCR	Receptor	4.8	3.85E-13
MYC	TF	4.8	3.86E-30
PRL	Cytokine	4.8	2.45E-16
NF-kB	TF	4.6	4.43E-6
TNF	Cytokine	4.6	1.12E-28

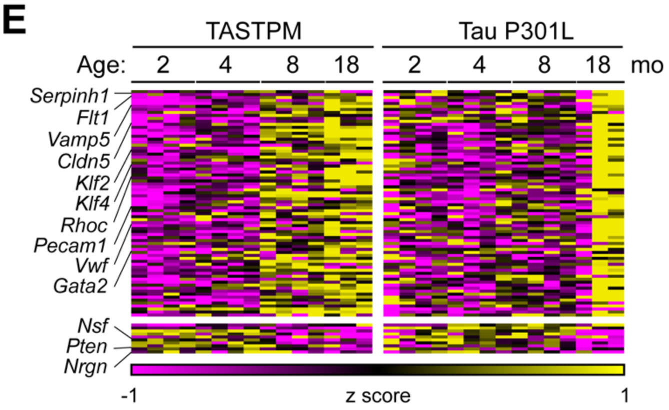

Figure S7. Endothelial cells contribute to immune dysregulation in tissues from patients with Alzheimer's disease (related to Figure 5)

(A) Changes in the proportion of endothelial (Endo) subpopulations (e1–e7) between Alzheimer's disease (AD) and normal control (NC) samples. (B) Only a few endothelial cell subpopulations contributed to the transcriptomic changes in AD. Bar plots showing the enrichment score for the AD-associated transcriptomic signature of each cell subpopulation. Up sig.: upregulated signature; Down sig.: downregulated signature. Enrichment scores were calculated by averaging the *z*-scores of individual genes in the transcriptomic signature (only positive values are shown). The dotted line indicates the cutoff of *z*-score = 0.5. (C) Bar plot showing the proportions of activated endothelial cells in AD and NC samples. (D) Ingenuity pathway analysis predicted the role of inflammation in stimulating endothelial activation in AD. The top 10 predicted upstream regulators are shown. TF: transcription factor. (E) Mouse models of amyloid-beta deposition and Tau hyperphosphorylation also exhibited transcriptomic reprogramming of endothelial cells similar to that observed in human AD samples. Heatmap showing the expression changes in differentially expressed genes (DEGs) in endothelial cells throughout disease progression in the TASTPM (model of amyloid-beta deposition) and Tau P301L (model of hyperphosphorylated Tau) mouse models of AD. All data are the mean ± SEM.
